# Symptomatic SARS-COV-2 reinfection: healthcare workers and immunosuppressed individuals at high risk

**DOI:** 10.1186/s12879-021-06643-1

**Published:** 2021-09-06

**Authors:** Efrén Murillo-Zamora, Xóchitl Trujillo, Miguel Huerta, Mónica Ríos-Silva, Felipe Aguilar-Sollano, Oliver Mendoza-Cano

**Affiliations:** 1 Departamento de Epidemiología, Unidad de Medicina Familiar No. 19, Instituto Mexicano del Seguro Social, Av. Javier Mina 301, Col. Centro, C.P. 28000 Colima, México; 2 Facultad de Medicina, Universidad de Colima, Av. Universidad 333, Col. Las Víboras, C.P. 28040 Colima, México; 3 Centro Universitario de Investigaciones Biomédicas, Universidad de Colima, Av. 25 de julio 965, Col. Villas San Sebastián, C.P. 28045 Colima, México; 4 Centro Universitario de Investigaciones Biomédicas, Universidad de Colima - Cátedras CONACyT, Av. 25 de julio 965, Col. Villas San Sebastián, C.P. 28045 Colima, México; 5 Programa de Maestría en Ciencias Médicas, Universidad de Colima, Av. Universidad 333, Col. Las Víboras, C.P. 28040 Colima, México; 6 Facultad de Ingeniería Civil, Universidad de Colima, km. 9 carretera Colima-Coquimatlán, Coquimatlán, C.P. 28400 Colima, México

**Keywords:** COVID-19, Severe Acute Respiratory Syndrome Coronavirus 2, Health Personnel, Risk Reduction Behavior

## Abstract

**Background:**

Knowledge regarding factors predicting the SARS-COV-2 reinfection risk is scarce and it has major implications in public health policies. We aimed to identify factors associated with the risk of symptomatic SARS-COV-2 reinfection.

**Methods:**

We conducted a nationwide retrospective cohort study and 99,993 confirmed cases of COVID-19 were analyzed.

**Results:**

The overall risk of reinfection (28 or more elapsed days between both episodes onset) was 0.21% (incidence density, 2.5 reinfections per 100,000 person-days) and older subjects and those with the mild primary disease were at reduced risk of the event. Healthcare workers and immunosuppressed or renal patients had at greater risk of SARS-COV-2 reinfection.

**Conclusions:**

If replicated in other populations, these results may be useful to prioritize efforts focusing on the reduction of SARS-COV-2 spread and the related burden.

## Background

The COVID-19 (coronavirus disease 2019) by SARS-COV-2 (severe acute respiratory coronavirus 2) pandemic is a complex phenomenon and reinfection is one of the many ongoing related debates [1]. Current knowledge regarding factors predicting the SARS-COV-2 reinfection risk is scarce and it has major implications in public health policies, including vaccination strategies and relaxation of social distancing measures [2].

The social and economic burden of the COVID-19 pandemic in Mexico has been high and by mid-February 2021, nearly 2 million laboratory-positive cases and nearly 170 thousand deaths had been registered [3]. Current vaccination efforts in Mexico started in late December 2020 and are slowly progressing; they first targeted health-care personnel directly involved in the attention of COVID-19 patients. Our study aimed to identify factors associated with the risk of SARS-COV-2 symptomatic reinfection in a large and nationwide cohort of laboratory-confirmed COVID-19 survivors.

## Methods

A nationwide and retrospective cohort study was conducted in Mexico including adults (aged 20 years or above) with laboratory-confirmed (quantitative reverse transcription polymerase chain reaction, RT-qPCR) COVID-19 by SARS-COV-2. This analysis took place in September 2020 and a broader description of the methods has already been published [4]. Adults whose symptoms appeared from March to June 2020 and who recovered to primary infection were analyzed.

The main binary outcome was symptomatic reinfection of SARS-COV-2 and was defined by the reappearance of symptoms of COVID-19 at 28 days or more after initial laboratory-confirmed illness [1] and a positive RT-qPCR result during second-time illness. Risk ratios (RR) and 95% confidence intervals (CI), calculated using generalized linear regression models, were used to identify factors associated with the risk of reinfection. All methods were performed following the relevant guidelines and regulations.

## Results

**Fig. 1:**
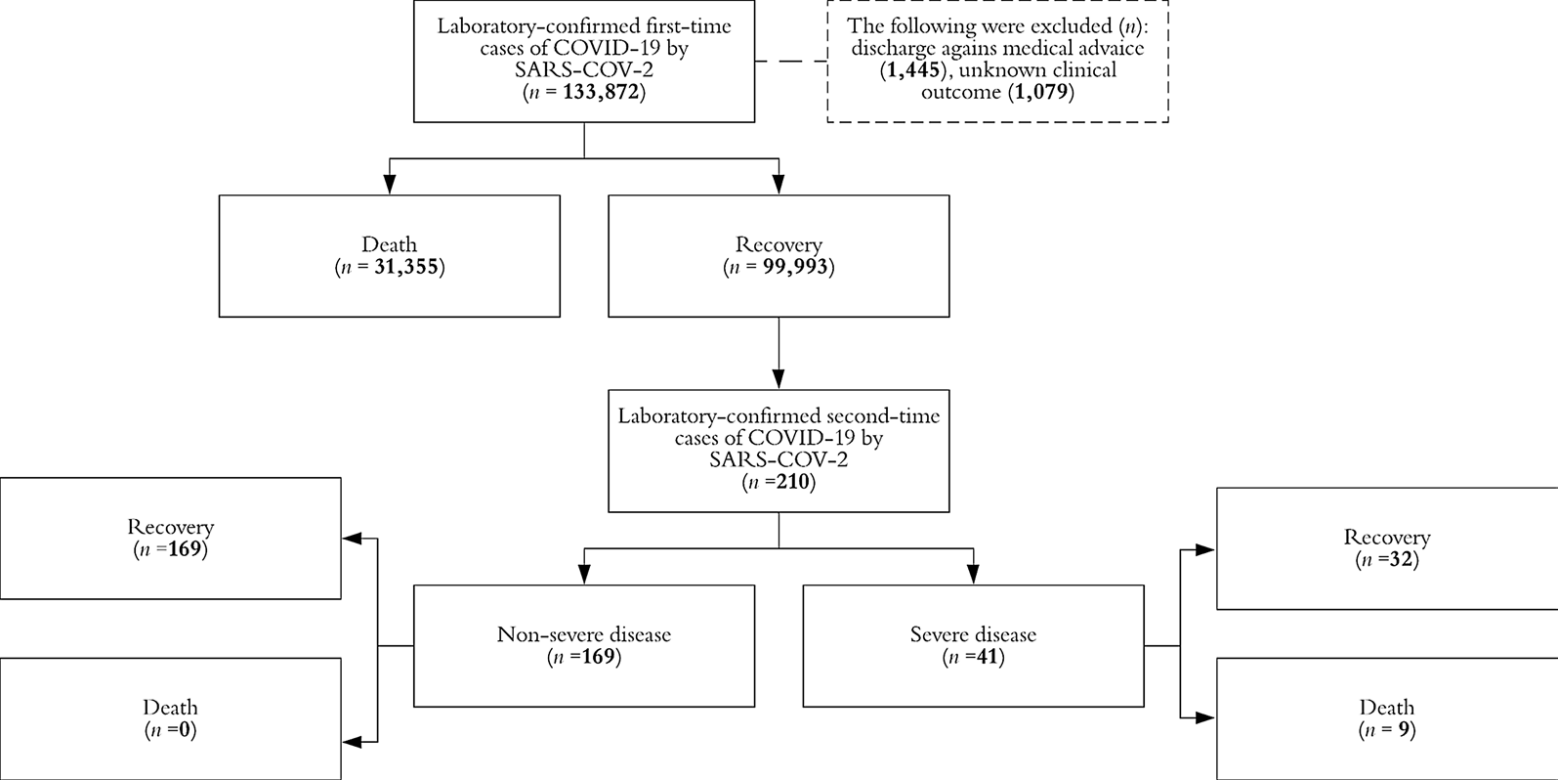
Study profile, Mexico 2020. *COVID-19* Coronavirus disease 2019, *SARS-COV-2* Severe acute respiratory syndrome coronavirus 2

Data from 99,993 participants were analyzed for a total follow-up of 8,268,237 person-days. The overall risk of SARS-COV-2 symptomatic reinfection was 0.21% (*n* = 210) and the incidence density was 2.5 reinfections per 100,000 person-days. The mean elapsed days (± standard deviation) between both COVID-19 episodes was 61.0 ± 31.0 and ranged from 28 to 116 days. Mild subsequent illness was documented in 169 patients (80.5%) of reinfected subjects and the observed fatality rate was 4.3% (*n* = 9). Figure 1 shows the study profile.

Table 1 shows the characteristics of the study sample according to the reinfection status for selected variables. Patients with SARS-COV-2 reinfection were younger and were more likely to be healthcare professionals or other related employments. They were also more likely to have had milder symptoms at primary disease and had a significantly higher prevalence of chronic kidney disease or immunosuppression (any cause except for type 2 diabetes mellitus or kidney disease).

**Table 1:** Characteristics of the study sample according to symptomatic SARS-COV-2 reinfection status, Mexico 2020

	Overall		SARS-COV-2 reinfection				*p*
			No		Yes		
	*n* = 99,993		*n* = 99,783		*n* = 210		
Gender							
Female	50,916	(50.9)	50,805	(50.9)	111	(52.9)	0.574
Male	49,077	(49.1)	48,978	(49.1)	99	(47.1)	
Age (mean ± SD, years)	42.2 ± 13.1		42.2 ± 13.1		39.2 ± 10.4		< 0.001
Age group (years)							
20–49	73,069	(73.1)	72,888	(73.1)	181	(86.2)	< 0.001
50–59	16,755	(16.8)	16,735	(16.7)	20	(9.5)	
60–69	6644	(6.6)	6638	(6.7)	6	(2.9)	
70 +	3525	(3.5)	3522	(3.5)	3	(1.4)	
Occupation							
Housewife	10,685	(10.7)	10,679	(10.7)	6	(2.9)	< 0.001
Healthcare worker	10,183	(10.2)	10,151	(10.2)	32	(15.2)	
Other healthcare-related	22,303	(22.3)	22,195	(22.2)	108	(51.4)	
Student	919	(0.9)	919	(0.9)	0	(0)	
Other	55,903	(55.9)	55,839	(56.0)	64	(30.5)	
Disease severity (at primary infection) ^*a*^							
Mild-moderate	81,018	(81.0)	80,827	(81.0)	191	(91.0)	< 0.001
Severe	18,975	(19.0)	18,956	(19.0)	19	(9.0)	
Personal history of:							
Obesity (BMI 30 or higher)							
No	81,531	(81.5)	81,353	(81.5)	178	(84.8)	0.228
Yes	18,462	(18.5)	18,430	(18.5)	32	(15.2)	
Type 2 diabetes mellitus							
No	86,909	(86.9)	86,718	(86.9)	191	(91.0)	0.082
Yes	13,084	(13.1)	13,065	(13.1)	19	(9.0)	
Arterial hypertension							
No	82,167	(82.2)	81,991	(82.2)	176	(83.8)	0.535
Yes	17,826	(17.8)	17,792	(17.8)	34	(16.2)	
Immunosuppression^b^							
No	98,830	(98.8)	98,627	(98.8)	203	(96.7)	0.003
Yes	1163	(1.2)	1156	(1.2)	7	(3.3)	
Chronic kidney disease							
No	98,440	(98.5)	98,239	(98.5)	201	(95.7)	0.001
Yes	1553	(1.5)	1544	(1.5)	9	(4.3)	
Chronic obstructive pulmonary disease							
No	98,872	(98.9)	98,667	(98.9)	205	(97.6)	0.083
Yes	1121	(1.1)	1116	(1.1)	5	(2.4)	
Asthma							
No	96,906	(96.9)	96,705	(96.9)	201	(95.7)	0.315
Yes	3087	(3.1)	3078	(3.1)	9	(4.3)	
Cancer (any site)							
No	99,744	(99.7)	99,535	(99.7)	209	(99.5)	0.508
Yes	249	(0.3)	248	(0.3)	1	(0.5)	

*SARS-COV-2* Severe acute respiratory coronavirus 2, *SD* Standard deviation, *BMI* Body mass index

1) The absolute and relative (%) frequencies are presented, except if the mean is specified; 2) *p*-value from chi-square or t-tests are presented as corresponding.

^a^Severe illness included the register of dyspnea requiring hospital admission.

^b^Immunosuppression referred to any cause of the related deficiency except for type 2 diabetes mellitus or renal impairment.

In multiple analyses (Table 2), increasing age was associated with a reduced risk of reinfection (RR_*per year*_ = 0.99997, 95% CI 0.99814–0.99958), as well as those with severe primary illness (RR = 0.9989, 95% CI 0.9981–0.9997). When compared with housewives, healthcare workers (RR = 1.0042, 95% CI 1.0030–1.0055) and other healthcare-related employees (RR = 1.0025, 95% 1.0012–1.0039) showed an increased reinfection risk. Other high-risk conditions included the personal history of immunosuppression (RR = 1.0038, 95% 1.0011–1.0065) or chronic kidney disease (RR = 1.0039, 95% CI 1.0016–1.0063).

**Table 2:** Predictors of symptomatic laboratory-confirmed SARS-COV-2 reinfection, Mexico 2020

	RR (95% CI), *p*					
	Bivariate analysis			Multiple analysis		
Gender						
Female	1.0000			1.0000		
Male	0.9998	(0.9993–1.0004)	0.574	1.0004	(0.9997–1.0009)	0.256
Age group (years)						
20–49	1.0000			1.0000		
50–59	0.9987	(0.9980–0.9995)	0.001	0.9989	(0.9981–0.9997)	0.006
60–69	0.9984	(0.9973–0.9996)	0.007	0.9984	(0.9972–0.9996)	0.009
70 +	0.9983	(0.9968–0.9999)	0.039	0.9982	(0.9966–0.9999)	0.032
Occupation						
Housewife	1.0000			1.0000		
Healthcare worker	1.0043	(1.0032–1.0054)	< 0.001	1.0042	(1.0030–1.0055)	< 0.001
Other healthcare-related	1.0026	(1.0013–1.0038)	< 0.001	1.0025	(1.0012–1.0039)	< 0.001
Student	0.9994	(0.9964–1.0025)	0.721	0.9993	(0.9961–1.0024)	0.646
Other	1.0006	(0.9996–1.0015)	0.227	1.0005	(0.9994–1.0016)	0.337
Disease severity (at primary infection)^a^						
Mild-moderate	1.0000			1.0000		
Severe	0.9987	(0.9979–0.9994)	< 0.001	0.9989	(0.9981–0.9997)	0.007
Personal history of:						
Obesity (BMI 30 or higher)						
No	1.0000			1.0000		
Yes	0.9996	(0.9988–1.0003)	0.228	0.9997	(0.9989–1.0004)	0.360
Type 2 diabetes mellitus						
No	1.0000			1.0000		
Yes	0.9993	(0.9984–1.0001)	0.082	0.9996	(0.9987–1.0005)	0.333
Immunosuppression^b^						
No	1.0000			1.0000		
Yes	1.0040	(1.0013–1.0066)	0.003	1.0038	(1.0011–1.0065)	0.005
Chronic kidney disease						
No	1.0000			1.0000		
Yes	1.0038	(1.0015–1.0061)	0.001	1.0039	(1.0016–1.0063)	0.001

*RR* Risk ratio, *CI* Confidence interval, *BMI* Body mass index

1) Generalized linear regression models were used to obtain RR and 95% CI; 2) Multiple regression coefficients were adjusted by variables listed in the table

^a^Severe illness included the register of dyspnea requiring hospital admission

^b^Immunosuppression referred to any cause of the related deficiency except for type 2 diabetes mellitus or renal impairment

## Discussion

Our results suggest that symptomatic SARS-COV-2 reinfection is a rare phenomenon and factors associated with its risk were characterized. However, these results must be carefully considered since currently there is not a well-defined criterion for SARS-COV-2 reinfection [1].

All enrolled subjects reported disappearance of symptoms from primary infection and the used cutout point to identify potential cases of reinfection (at least 28 days between both laboratory-positive episodes) seemed to be epidemiologically useful since is according to the observed IgG antibodies titers decay in recovered COVID-19 patients [5]. In our study, no PCR testing was performed to avoiding the inclusion of potential cases of persistent viral shedding.

However, and despite this later, the computed incidence density in our analysis was considerable lower (2.5 vs. 7.6 reinfections per 100,000 person-days) than that estimated in a large cohort study where PCR and antibodies testing were available [6]. Therefore, the criteria proposed by Tomassini et al. [1] and which was used in our study may be particularly relevant to identify reinfection cases in sources limited healthcare settings, where no genetic sequencing of viral strains are systematically performed.

According to our findings, healthcare workers and other related employees (i.e. medical assistants, dentists, etc.) are at increased risk of SARS-COV-2 symptomatic reinfections, which sounds plausible given the increased risk of exposure among these subjects [7]. Similar findings were recently observed in a cohort that took place in two cities in the USA [8].

Mild COVID-19 patients at primary episodes also may be at greater risk of reinfection, which may be secondary to lower antibodies titers when compared with pneumonia patients [9]. The association between immunosuppression and [2] renal impairment with COVID-19 risk has been widely discussed [4, 10].

If later replicated, further research is needed to identify factors determining a decreased reinfection risk among older participants and after adjusting by multiple exposures. We hypothesize that a reduced COVID-19 awareness among younger subjects may be implied, at least partially. Besides, longer isolation after the first episode among older subjects and those with more severe disease may be determining the observed scenario.

## Conclusions

To the best of our knowledge, this is the first study evaluating predictors of symptomatic SARS-COV-2 reinfection in a large subset of individuals and populations at high-risk were identified. Clinical and epidemiological research regarding SARS-COV-2 reinfection has immediate implications for public health policies focusing on the reduction of viral spread.

## Data Availability

The datasets generated during and/or analyzed during the current study are available from the corresponding author on reasonable request.

## Abbreviations

COVID-19: Coronavirus disease 2019
SARS-COV-2: Severe acute respiratory coronavirus 2
RT-qPCR: Quantitative reverse transcription polymerase chain reaction
RR: Risk ratio
CI: Confidence interval
